# Planning considerations prior to laryngectomy for a patient infected with severe acute respiratory syndrome coronavirus-2 pre-operatively

**DOI:** 10.1017/S0022215120002388

**Published:** 2020-11-04

**Authors:** H Coleman, T Tikka, S Okhovat, S K Kang

**Affiliations:** ENT Department, University Hospital Monklands, Airdrie, Scotland, UK

**Keywords:** Coronavirus, COVID-19, Laryngectomy, Squamous Cell Carcinoma

## Abstract

**Background:**

Coronavirus disease 2019 was declared a pandemic on 11th March 2020. All non-urgent surgical procedures have been postponed indefinitely. The British Association of Head and Neck Oncology state that only those with treatable head and neck cancer unsuitable for alternative treatment should undergo surgery. This paper details our management of a patient who tested positive for severe acute respiratory syndrome coronavirus-2 days before curative surgery for laryngeal cancer.

**Case report:**

By following British Association of Head and Neck Oncology guidance, a 49-year-old male scheduled for total laryngectomy and bilateral neck dissection for a T_3_ transglottic squamous cell cancer was pre-operatively identified as an asymptomatic carrier of severe acute respiratory syndrome coronavirus-2. Following 14-day isolation and laboratory proven viral clearance, he underwent successful major surgery. He was managed throughout the peri- and post-operative phases without complications or adverse effects on staff.

**Conclusion:**

With careful planning, previous coronavirus disease 2019 positive status should not prevent an individual from undergoing successful total laryngectomy and bilateral neck dissection in a safe and timely manner during the pandemic.

## Introduction

Coronavirus disease 2019 (Covid-19) is the disease caused by the respiratory virus severe acute respiratory syndrome coronavirus-2 (SARS-CoV-2), responsible for the global pandemic declared by the World Health Organization on 11th March 2020. The disease is associated with severe morbidity and mortality in the elderly, the immunocompromised, and those with co-morbidities such as hypertension, diabetes and obesity. Coronavirus disease 2019 has claimed the lives of 852 758 people as of 2nd September 2020.^1,2^

Although the exact transmission route of the virus remains unclear, it has been well documented that ENT surgeons and intensivists are at greatest occupational risk given the requirement of examinations, instrumentation and aerosol-generating procedures (AGPs) involving the nose and throat, which are known to harbour a high viral load.^3^ During the Covid-19 pandemic, all non-urgent surgical procedures have been postponed indefinitely. Life and limb saving surgery, including operations for cancer, should go ahead.^4^ With regard to head and neck cancer, guidelines published by the British Association of Head and Neck Oncologists state that only those with treatable disease not suitable for alternative treatment (i.e. radiotherapy) should undergo laryngectomy for laryngeal or hypopharyngeal squamous cell carcinoma (SCC) given the high risk of viral transmission during surgery.^5^

Although all cancer centres have been operating as appropriate throughout the Covid-19 pandemic, there are no descriptions of patients having been previously infected with SARS-CoV-2 and successfully undergoing major surgery. Here we present the case of a 49-year-old male who underwent successful total laryngectomy and bilateral neck dissection for a T_3_ transglottic SCC who tested positive for SARS-CoV-2 in the pre-operative period. We detail his pre- and peri-operative management, post-operative course, and safe discharge.

## Case report

A 49-year-old male was referred to the head and neck service with a 6-week history of hoarseness. His past medical history included anxiety and hypertension. He lived alone, smoked 15 roll-up cigarettes daily and consumed a moderate volume of alcohol per week. He had a World Health Organization performance status score of 1 and a Malnutrition Universal Screening Tool score of 0. Direct examination and subsequent computed tomography of the head and neck confirmed a T_3_ transglottic lesion with no evidence of distant disease; biopsy of this lesion confirmed a moderately differentiated SCC. His case was discussed at the regional head and neck multidisciplinary team meeting, and a total laryngectomy and bilateral neck dissection was proposed, to which he subsequently agreed.

His operation was planned for the end of March 2020 for social reasons; however, two weeks prior to his operation date the Covid-19 pandemic was declared. The patient underwent standard pre-operative investigations including blood work, electrocardiography and an echocardiogram, the findings for all of which were normal. British Association of Head and Neck Oncology guidance for laryngectomy during Covid-19 was followed; the patient was advised to self-isolate for 14 days and asked to attend his local ENT department for two Covid-19 swab tests within the week prior to surgery. His first swab was negative; however, the second swab, performed 4 days later, was positive for SARS-CoV-2. His surgery was postponed and he was asked to self-isolate for a further 14 days. He was asymptomatic at the time of testing and remained so throughout his isolation period.

During the 14-day self-isolation period, he underwent Covid-19 swab testing on days 7 and 14. Both swabs were negative and therefore his operation was rescheduled to take place 2 days following his most recent negative swab test. The day before surgery, he was admitted to a ‘clean’ tertiary centre with no accident and emergency or dedicated Covid-19 ward. He was again swab tested on admission and found to be negative.

The patient successfully underwent total laryngectomy and bilateral neck dissection following clearance of Covid-19, only two weeks later than initially planned. Despite his negative swab results, enhanced personal protective equipment (PPE) was worn throughout the surgical procedure by operating theatre staff, including a filtering facepiece code 3 (FFP3) mask, face shield, disposable cap, fluid-resistant gown and gloves. There were no surgical or anaesthetic-related peri-operative complications.

The patient progressed well on the ward following surgery. Healthcare professionals wore enhanced PPE, including FFP3 masks, face shield, fluid-resistant gowns and gloves when carrying out AGPs (e.g. suctioning and stoma care). On morning ward rounds, he was reviewed by a senior member of the medical team who wore a fluid-resistant surgical mask, face shield, apron and gloves. There were no post-operative complications and the patient was safely discharged two weeks after his surgery.

The patient was reviewed in clinic one month post-operatively; his wounds were healing well and he was eating and drinking without issue. His final pathology report revealed a completely excised, moderately to poorly differentiated laryngeal SCC, pathologically confirmed as tumour–node stage T_3_N_0_ (based on the 8th edition of the Union for International Cancer Control tumour–node–metastasis (TNM) classification). Following discussion with the oncology department, the patient decided against adjuvant radiotherapy. The British Association of Head and Neck Oncology advises against same-day tracheoesophageal puncture and speech valve insertion during the pandemic, to reduce the length of hospital stay and associated complications; he therefore remains on the waiting list for this.

## Discussion

As a result of the declaration of the Covid-19 pandemic immediately following the decision to proceed with this patient's curative surgery, the standard pre-operative assessment required alteration, to ensure both patient and staff safety. British Association of Head and Neck Oncology guidance for laryngectomy during the Covid-19 pandemic was adhered to.^5^ Coronavirus disease 2019 testing must be performed before tracheostomy and certainly before laryngectomy; two Covid-19 tests over an undefined interval period provide more definitive results. A laryngectomy must not be performed in a Covid-19 positive patient. In cases of airway obstruction, intubation or tracheostomy must be performed as an alternative, and definitive treatment performed once the patient is Covid-19 negative. All patients, regardless of Covid-19 status, must be managed peri-operatively on the assumption they are infected; enhanced PPE and FFP3 masks must be worn by all operating theatre staff. Primary tracheoesophageal puncture is not recommended, to avoid associated risks in the immediate and short-term recovery period. Surgery with greater post-operative risks, such as microvascular free flaps and pedicled flaps, should be avoided if possible.

By following British Association of Head and Neck Oncology guidance, we were successful in identifying an asymptomatic Covid-19 carrier in the pre-operative phase. We were then able to advise a 14-day self-isolation period, to ensure his safety and that of other patients and healthcare staff.

**Fig. 1.**
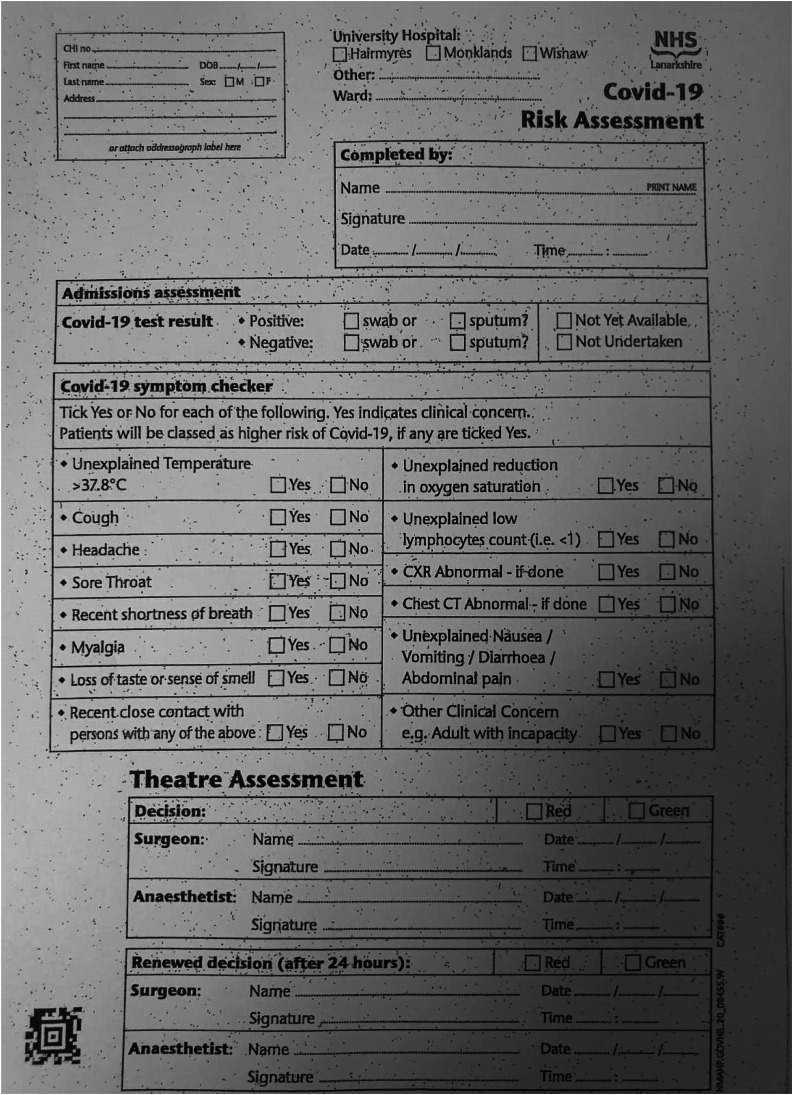
NHS Lanarkshire coronavirus disease 2019 (Covid-19) operating theatre risk assessment form. CXR = chest X-ray; CT = computed tomography

In this case, two consecutive negative tests separated by 7 days during the 14-day self-isolation period facilitated prompt and safe operating theatre scheduling for a patient who required urgent major surgery during the pandemic. We found that one further negative swab on the day prior to surgery provided additional reassurance of a low transmission risk. Within our institution, we have also implemented a Covid-19 pre-operative safety checklist to be completed by a member of the surgical and anaesthetic teams on the morning of surgery (Figure 1). Patients should be clinically assessed as standard on the day of surgery, regardless of Covid-19 status, by the list anaesthetist and operating surgeon, regarding fitness to proceed with the proposed procedure.

The enhanced PPE protocol in the peri- and post-operative periods should be adhered to by all involved clinical staff, regardless of known Covid-19 status, given the unknown false negative Covid-19 polymerase chain reaction testing rates.^6^ In the case of the post-laryngectomy patient, the minimum of a surgical fluid-resistant face mask, face shield, apron and gloves should be worn for daily post-operative examination, performed by the most senior medical staff member, to allow the identification of complications at the earliest opportunity. Irrespective of Covid-19 status, enhanced PPE including FFP3 masks should be worn when performing AGPs, including suctioning of the laryngectomy stoma. All clinical staff should perform thorough hand hygiene before and after any patient contact, and at regular intervals throughout the working day.

Coronavirus disease 2019 testing in the post-operative period should be based on clinical suspicion for SARS-CoV-2 infection. Our local practice requires swab testing every 4 days for all in-patients aged over 70 years.

- During the coronavirus disease 2019 (Covid-19) pandemic, all non-urgent surgical procedures have been postponed indefinitely
- Only those with treatable head and neck cancer unsuitable for alternative treatment should undergo surgery given the high-risk nature of the procedure
- British Association of Head and Neck Oncology has produced guidance on managing patients undergoing laryngectomy during the pandemic
- This is the first reported case of a patient identified as Covid-19 positive pre-operation successfully undergoing total laryngectomy and bilateral neck dissection
- This treatment was conducted following a 14-day self-isolation period and viral clearance
- If guidance is followed, previous Covid-19 positive status should not prevent laryngectomy and neck dissection in a safe and timely manner during the pandemic

It has been previously described that delays of four weeks in those with moderately to poorly differentiated laryngeal SCC risked worsening TNM stage and prognosis.^7^ In this case, the two-week delay for surgery did not appear to affect outcome in terms of pathology and therefore patient prognosis. Despite this good outcome following a two-week delay, surgery of this nature should be performed as soon as is safely possible.

## Conclusion

This is the first report in the literature of a patient having been identified as Covid-19 positive in the pre-operative period and subsequently undergoing successful total laryngectomy and bilateral neck dissection after viral clearance and a 14-day isolation period. This case therefore proves that if the appropriate steps are taken in the pre-, peri- and post-operative phases, then previous Covid-19 positive status should not prevent an individual from undergoing successful surgery for laryngeal cancer in a safe and timely manner during the Covid-19 pandemic.
